# Experimental demonstration of multiple Fano resonances in a mirrored array of split-ring resonators on a thick substrate

**DOI:** 10.1038/s41598-022-20434-x

**Published:** 2022-09-23

**Authors:** Andrius Kamarauskas, Dalius Seliuta, Gediminas Šlekas, Modestas Sadauskas, Evaldas Kvietkauskas, Romualdas Trusovas, Karolis Ratautas, Žilvinas Kancleris

**Affiliations:** 1 Center for Physical Sciences and Technology, Savanoriu Ave. 231, 02300 Vilnius, Lithuania; 2 Vilnius Gediminas Technical University, Sauletekio Ave. 11, 10223 Vilnius, Lithuania

**Keywords:** Optical materials and structures, Metamaterials, Surface patterning

## Abstract

This work demonstrates the first experimental observation of multiple Fano resonances in the terahertz range in a system based on an array of mirror-symmetric split-ring resonators deposited on low-loss and low-refractive index polytetrafluoroethylene (PTFE) substrate. For the first time, selective surface activation induced by laser technology has been used to deposit a copper layer on a PTFE substrate with the further application of standard mask lithography for metasurface manufacturing.

## Introduction

Fano-type resonances are observed in metasurfaces made of split-ring resonators (SRR). In order to obtain it, additional asymmetry is introduced in the SRR. Typically, the ring is split into two sections of different lengths where the so-called "dark mode" is excited, responsible for the appearance of the Fano resonance^1^. Due to the weak coupling of the dark mode with the external electric fields, the Fano resonance demonstrates a high resonance quality. Therefore, it is expected that such a metamaterial might find application in developing a variety of sensors^2^.

Because of the different application requirements, research interest in the Fano resonance field has spread from a single Fano resonance to multiple Fano resonances. Multispectral Fano resonances are promising in multichannel biochemical sensing^3^, multi-band second harmonic generation^4^, and multi-band absorbers/emitters^5^. While single Fano resonance arises from combining one bright mode and one dark mode, combining a bright mode with several dark modes can result in several Fano resonances. Multiple Fano resonances are created by introducing new asymmetries into a planar periodic structure^6^, collective excitation of a metamolecule lattice consisting of two different metamaterial resonators^7^, by coupling between the surface plasmon-polariton mode and multi-order planar waveguide modes^8^. Multiple Fano resonances in metal–insulator-metal waveguide structures with different shapes of cavities^9^ have attracted the attention of many researchers due to their outstanding features, including ease of integration and deep subwavelength confinement of light in the visible and near-infrared wavelengths. Hybrid metamaterial waveguide (HMW) structures have been proposed to establish multiple Fano peaks caused by destructive interference of dark quasi-guided and bright plasmon modes. Theoretical considerations have shown that owing to the multimode characteristics of the slab waveguide, HMW design can offer an easy way to realize multiple Fano resonances in simple metal resonators operating in the far infrared and terahertz spectral ranges^10–12^. Recently in the GHz frequency range, a multiple electromagnetically induced transparency using a double-layered metasurface^13^ and ultra-wideband polarization conversion extension using multiple Fano resonances^14^ have been demonstrated experimentally. In both cases, to achieve multiple resonances, the unit cells of proposed metasurfaces have been rather complicated.

In this work, we present the first experimental observation of multiple Fano resonances in the terahertz range in HMW system based on an array of mirror-symmetric split-ring resonators^15,16^. We propose a scheme for multiple Fano resonance observation via interaction of plasmonic mode with dielectric waveguide modes appearing in a mirror-symmetric array of SRRs deposited on low-loss and low-refractive index polytetrafluoroethylene (PTFE) substrate. By increasing the substrate's thickness, higher waveguide modes are excited. As a result, they interact with plasmonic mode, and multiple Fano resonances appear. The number of Fano resonances and their characteristic frequencies can be simply adjusted by changing the thickness of the substrate. Remarkably, our design provides an open (cladding-free) waveguide with great potential for designing multi-wavelength biosensors, refractive-index sensors, and filters.

Several methods are known that can be applied to fabricate metasurfaces, such as inkjet printing^17^, screen printing^18^, roll-to-roll printing^19^, chemical vapor deposition^20^. However, none of the mentioned methods can deposit a metal layer on the PTFE substrate with sufficient adhesion to the substrate. Therefore, the novel selective surface activation by laser (SSAIL) method is used in this work^21–24^. SSAIL contains 3 main steps: laser modification of the dielectric surface, chemical activation of the modified areas by dipping into solution and chemical electroless metal deposition of the activated parts. The new technology offers laser writing speeds of up to 4 m/s, and herewith spatial plating pitch is kept as narrow as 25 µm. Compared with other plating technologies, the main advantage of the SSAIL process is that process is selective, and copper deposition appears only on the laser-modified surface. Moreover, SSAIL does not require special additives in the polymer matrix, and standard commercial material (available on the market) can be used as a circuit carrier. SSAIL brings unique benefits for PTFE application by forming high adhesion of copper to the substrate. Further, the SRR structures are fabricated using standard mask photolithography.

## Results and discussion

### Experimental setup

**Figure 1:**
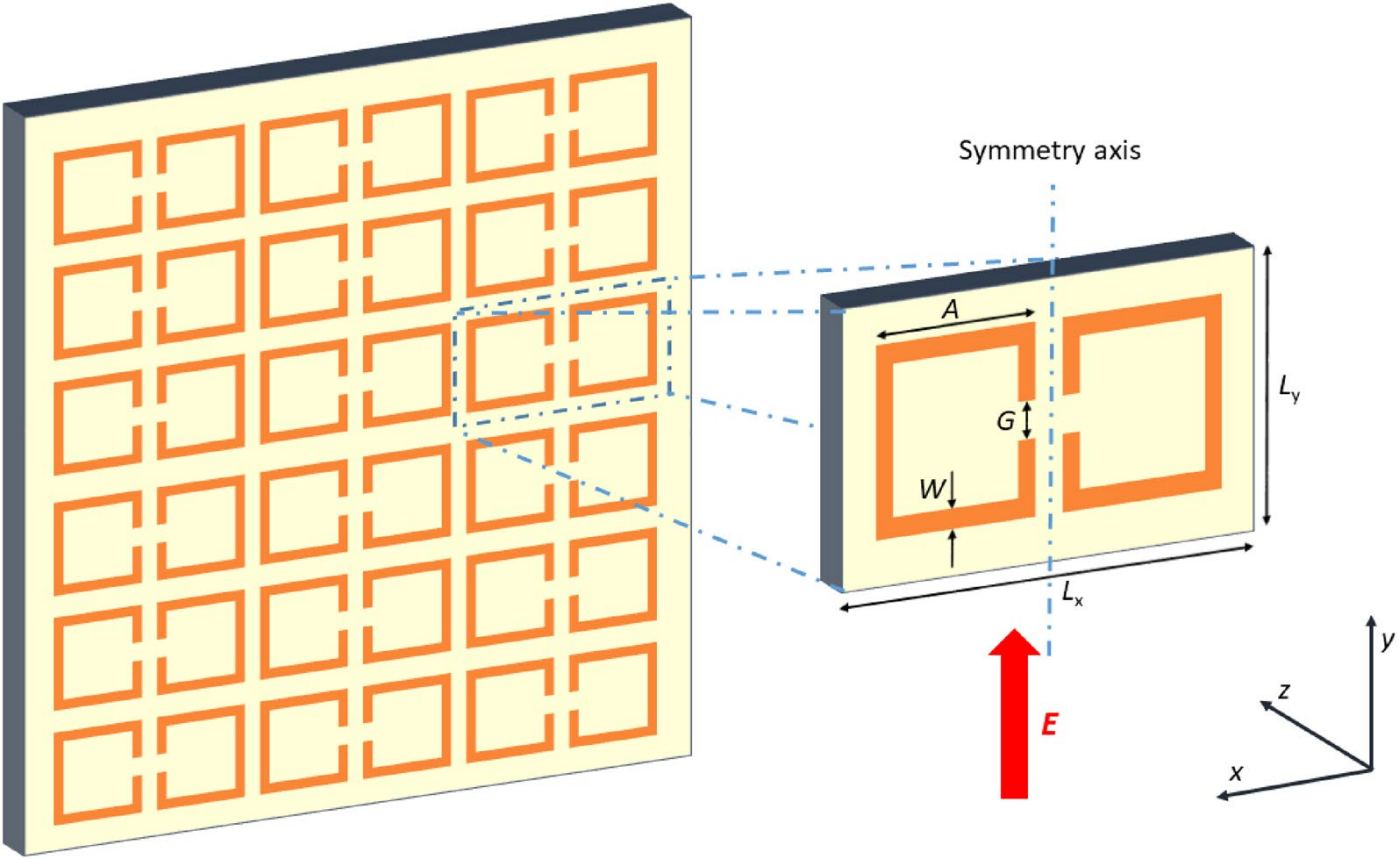
Schematics of the mirrored array of SRRs and enlarged single unit cell. Dimension of the unite cell in *x* direction *L*_*x*_ is two times larger than *L*_*y*_ = 600 μm. The dimensions of SRR are *A* = 500 μm, *W* = *G* = 50 μm, the metallization thickness is 10 μm, and the external electric field is parallel to the *y* axis.

The metasurface we study is schematically shown in Fig. 1. It is seen that the SRR, in every second column of the array, is rotated by 180 degrees. Such a configuration has allowed the detection of single Fano resonance earlier^15,16^.

Since the electric field of electromagnetic waves crosses the gap of SRR, odd plasmonic modes (*n* = 1, 3, …) are excited in them, where *n* is the number of half wavelengths of oscillations fitting in the perimeter of SRR^25^.

For the experimental investigation, the resonators are formed on a polytetrafluoroethylene (PTFE) substrate. PTFE is a material having unique mechanical and electrical properties. Its distinctive feature is low dielectric losses. For this reason, PTFE is an attractive material for applications in the GHz band. However, since the material has a very low coefficient of friction, it is challenging to deposit the metal on the surface of the PTFE. There are several studies based on chemical etching and plasma etching^26,27^. However, we apply a relatively new, SSAIL technology proposed in^21^. SSAIL technology has already been applied for the metallization of different dielectric materials, including glass, ceramics, and various polymers. In this work, we combine chemical and laser technologies, and demonstrate the first application of this method for PTFE metallization with sufficient adhesion and outstanding electromagnetic properties in THz range (see Methods).

### Numerical modeling

The numerical simulations are performed using a custom-made program based on a finite-difference time-domain method. For the simulation of the SRR array, the unit cell is shown in Fig. 1 is used. The differentiated Gaussian pulse is generated using a total-field–scattered-field plane wave source, and the modelling domain is truncated by the uniaxial perfectly matched layers to introduce the absorption of waves without reflection^28^. Finally, the method based on the generalized Goertzel algorithm is used to calculate the transmittance spectra^29^.

The PTFE layer on which SRRs are deposited can be considered a dielectric waveguide. Due to the diffraction of electromagnetic waves on a periodic array of SRRs (ref to Fig. 1), the waveguide modes can be excited in it. Reflecting on the sample's front and rear planes, they are traveling in x0y plane. To determine them, one has to use the techniques described in^30,31^. The diffracted beam is trapped in a dielectric plate when it falls on the dielectric-air interface at an angle greater than the total internal reflection angle *θ*_*c*_, where

1 $$ \sin \theta_{c} = 1/\sqrt \varepsilon $$

To find the modes propagating through a dielectric slab, one has to solve an algebraic equation relating the electromagnetic wave components on both sides of the interface^30^ or satisfy the so-called "self-consistency condition" for the beam propagating through the dielectric and reflecting from both interfaces^31^. In both cases, the solution has to be found numerically. Usually, the zero-mode in a dielectric waveguide has no critical frequency, while the higher modes have. Therefore, they can only be excited at a frequency above the critical one. Since, in our case, the modes in the dielectric substrate are excited by the diffraction of a ray from a periodic pattern formed on the surface of the dielectric, the angle *θ*_*d*_, for the wave incident perpendicularly to the metasurface, can be expressed as

2 $$ \sin \theta_{d} = \frac{\lambda l}{{\sqrt \varepsilon L_{x} }}, $$

here *λ* is the wavelength of the incident rays on the metasurfaces, *l* = 1, 2, … is the diffraction order, and *L*_*x*_ denotes the period of the resonator array in the x-direction (ref to Fig. 1). As far as the external field is directed in the *L*_*y*_ direction, we consider only TE modes excited in the dielectric substrate. Furthermore, from the infinitely large number of possible modes which could be excited in the dielectric waveguide, only those whose angle of incidence on the interface corresponds to the angle of the diffracted beam are considered.

## Discussion

**Figure 2:**
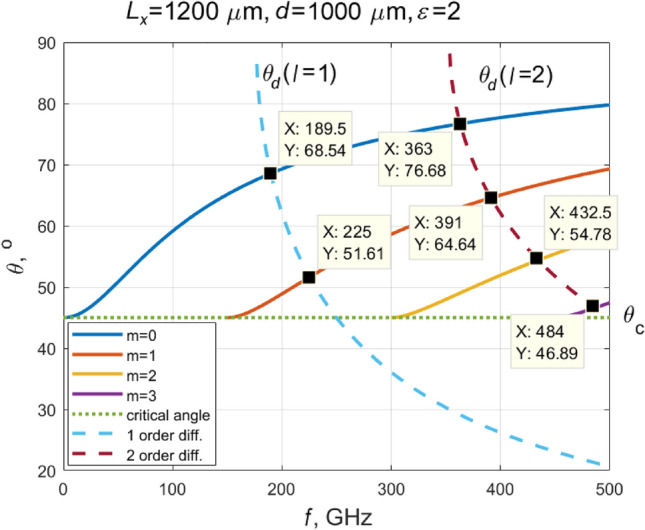
Dependences of the angle, at which light impinges on the dielectric-air interface, on frequency for a dielectric plate in the air. The thickness of the plate *d* = 1000 μm, its dielectric constant is 2.0, and the grating period *L*_*x*_ = 1200 μm. Solid lines show allowed modes in the plate, dashed lines demonstrate diffraction angles, and the dotted line denotes the angle of total internal reflection. The intersection points of solid and dotted lines show modes that can be excited in the plate with a metasurface deposited on its surface, the periodicity of which is *L*_*x*_.

In Fig. 2, we present an example of modes that can be excited in a 1000 µm thick substrate with 1200 µm period grating deposited on its surface. The solid lines show the angle of incidence on the dielectric-air interface of the excited waveguide modes in a dielectric plate surrounded by air. The angle of incidence *θ* at the lowest frequency for any mode is equal to the critical angle, which is indicated by the horizontal dotted line in the figure. The dashed lines show the angles at which the beam is deflected by diffraction from a periodic structure deposited on the surface of the plate. Obviously, the intersection of the dashed and solid curves shows the modes that can be excited in a plate with a periodic metasurface. In this particular case, the six modes can be excited. Two of them (m = 0, 1) appear due to the first-order diffraction, and four—second order diffraction (m = 0–3). Their frequencies and angles *θ* are shown in Fig. 2.

**Figure 3:**
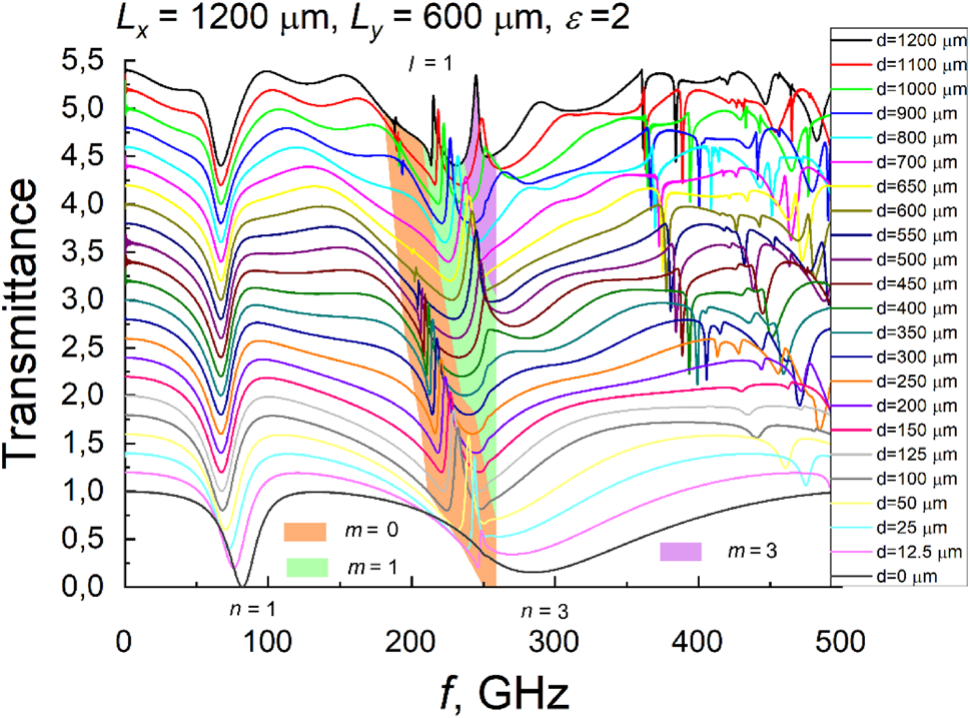
Calculated transmittance spectra of metamaterial composed of a mirrored array of split-ring resonators on a different thickness of the substrate (ε = 2). Substrate thickness is denoted in the figure. For the sake of clarity, spectra are shifted in the ordinate axis. Letter *n* marks plasmonic mode number, different colors correspond to different waveguide mode numbers, and letter *l* stands for the diffraction order.

The calculated frequency dependences of the transmittance of the mirrored arrays formed on different thicknesses of a substrate are shown in Fig. 3. For clarity, the curves are shifted in the ordinate axis with respect to one another. As can be seen from the figure, the Fano resonance observed previously^15,16^ in arrays formed on a relatively thin substrate, shifts towards the lower frequencies with increasing substrate thickness. The first-order plasmonic resonance behaves similarly. However, as the thickness of the substrate increases, instead of one Fano-type resonance, two, and in samples on the thickest substrate, even three clear Fano resonances can be distinguished.

**Figure 4:**
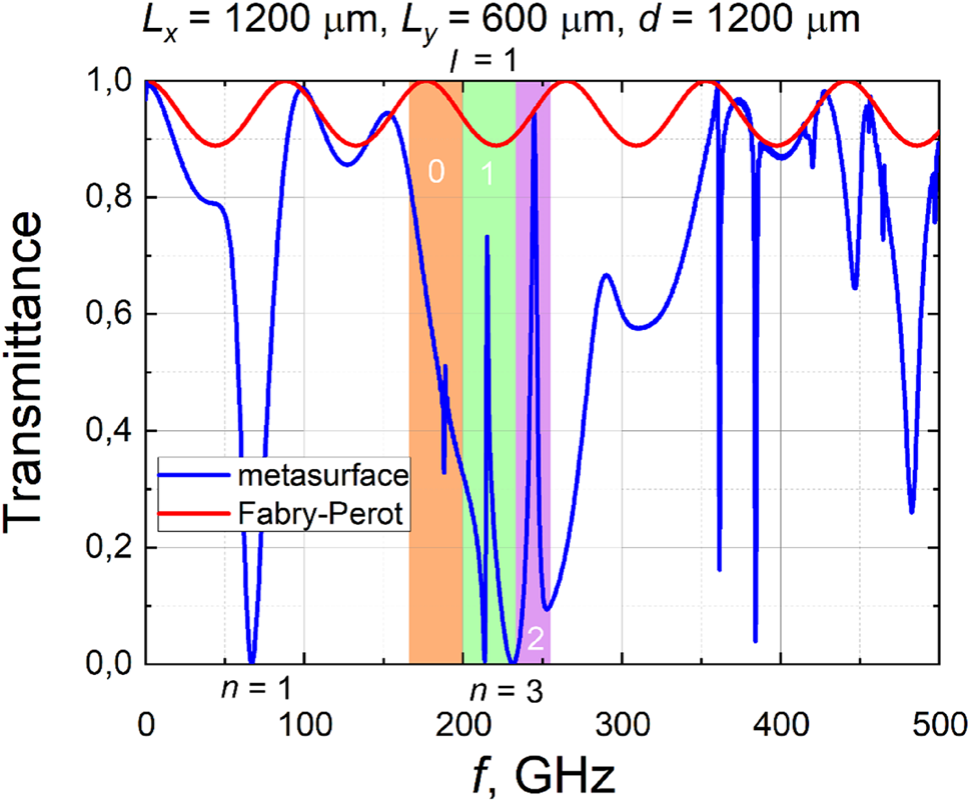
Metasurface transmittance spectrum and Fabry–Perot spectrum fringes for the thickest considered substrate. Letter *n* denotes plasmonic mode number, different colors correspond to different waveguide modes, numbers in a colored background mark waveguide mode, and letter *l* stands for diffraction order.

As we have studied metasurfaces formed on thick substrates, they should also exhibit Fabry–Perot resonances. However, due to the low dielectric permittivity of the substrate, the depth of the bandwidth modulation caused by these resonances is not large. This is confirmed by the computational results shown in Fig. 4. In addition to the calculated spectrum for a metasurface formed on a 1.2 mm thick substrate, the Fabry–Perot resonance-mediated bandwidth modulation is shown. It is on the order of 10%, and its influence on the metasurface transmittance is not significant in the frequency range where sharp Fano resonances are observed.

**Figure 5:**
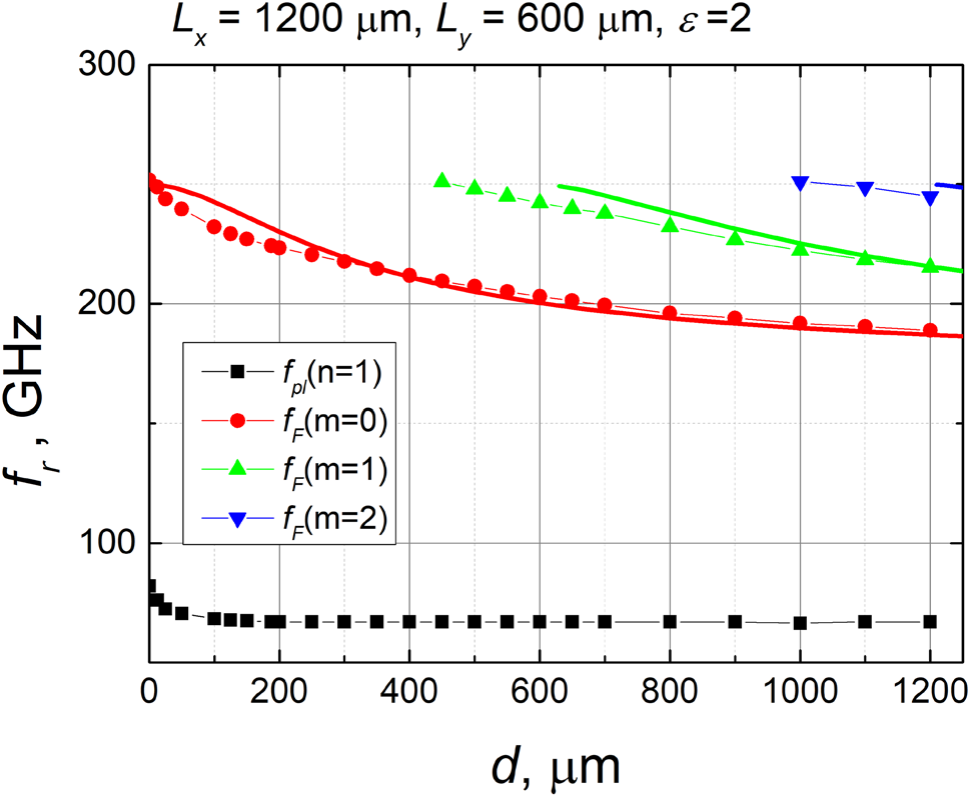
Dependences of resonance frequencies on the thickness of the substrate. Points show results extracted from the calculated transmittance spectrum. Squares correspond to the first plasmonic resonance, and other points show Fano resonance frequencies. Solid lines demonstrate frequencies of waveguide modes excited in a dielectric substrate.

From the calculated spectra, shown in Fig. 3, we determined the frequency dependence of the Fano resonance and the first plasmonic resonance on the substrate thickness. Symbols in Fig. 5 show these results. As Fig. 5 shows, the plasmonic resonance frequency decreases with the increasing thickness of the dielectric substrate until the thickness reaches about 100 μm. Further increase of thickness does not influence plasmonic frequency. One can assume that for d > 100 μm, the effective dielectric permittivity of the interface can be expressed as the average of the dielectric permittivities on either side of the interface ${\varepsilon }^{*}=(\varepsilon +1)/2,$ where *ε* is the permittivity of the dielectric substrate, and unity corresponds to the relative dielectric permittivity of free space. Considering plasmonic resonance as the resonance of the LC circuit, it is clear that by increasing *d* we are changing the capacitance of the equivalent circuit, whereas inductance remains unchanged. Therefore, formally the dependence of plasmonic resonance frequency on ε^*^ can be expressed ${f}_{pl}=1/\left(2\pi \sqrt{LC}\right)\sim \frac{1}{\sqrt{{\varepsilon }^{*}}}$. Having in mind that *ε* = 2, one can get that the resonance frequency should decrease by a factor of 1.225 when *d* is increasing. Surprisingly, this is precisely the same as the ratio obtained from the simulation results: *f*_*pl*_(*d* = 0)/ *f*_*pl*_(*d* > 100 μm) = 82/67 = 1.224.

As seen from Fig. 5, collected from spectral dependencies of transmittance (ref to Fig. 3), Fano resonance frequencies demonstrate a much stronger dependence on the substrate thickness than the first plasmonic resonance. Consequently, it can hardly be explained by the variation of the effective dielectric permittivity. However, a periodic array of SRRs deposited on the dielectric leads to diffraction of electromagnetic radiation, and diffracted rays falling on the substrate at an angle larger than the angle of total internal reflection cannot escape from the substrate. Thus, waveguiding modes are excited in the dielectric, which interacts with the plasmonic resonance (*n* = 3) giving rise to the Fano resonances mentioned above.

Considering only the first diffraction order, as it is of interest in the frequency range up to 300 GHz, where Fano type resonances have been observed, we have calculated the dependence of the excited zeroth, first and second-order waveguide mode frequencies on the thickness of the substrate. The results of the calculation are shown in Fig. 5 by solid lines. It is seen that Fano resonance frequencies pretty well coincide with waveguide mode frequencies, especially when the thickness of the substrate increases. This fact strongly supports the proposition that multiple Fano resonances appear as a consequence of the interaction of a wide plasmonic mode with a narrow waveguide modes. There is some discrepancy in the calculated mode frequencies compared with the data obtained from the simulated spectra. The point is that the modes are calculated for a dielectric plate, both sides of which are surrounded by air. In the actual situation, one side of the plate is covered by metallic SRRs, which causes the show of plasmonic modes and Fano-type resonances in transmission spectra. This obviously results in a change in the wave's phase reflected from the metasurface that may influence the excited waveguide mode frequency^32^. It is apparent that the difference between the results obtained from the mode approximation and from the spectral simulations decreases with the increasing substrate thickness. This happens since the angle of incidence to the interface increases with increasing *d*, and, therefore, the additional phase shift due to the metallic lattice has a relatively smaller and smaller contribution to the total phase shift accumulated by the beam traveling through the sample as a dielectric waveguide mode.

**Figure 6:**
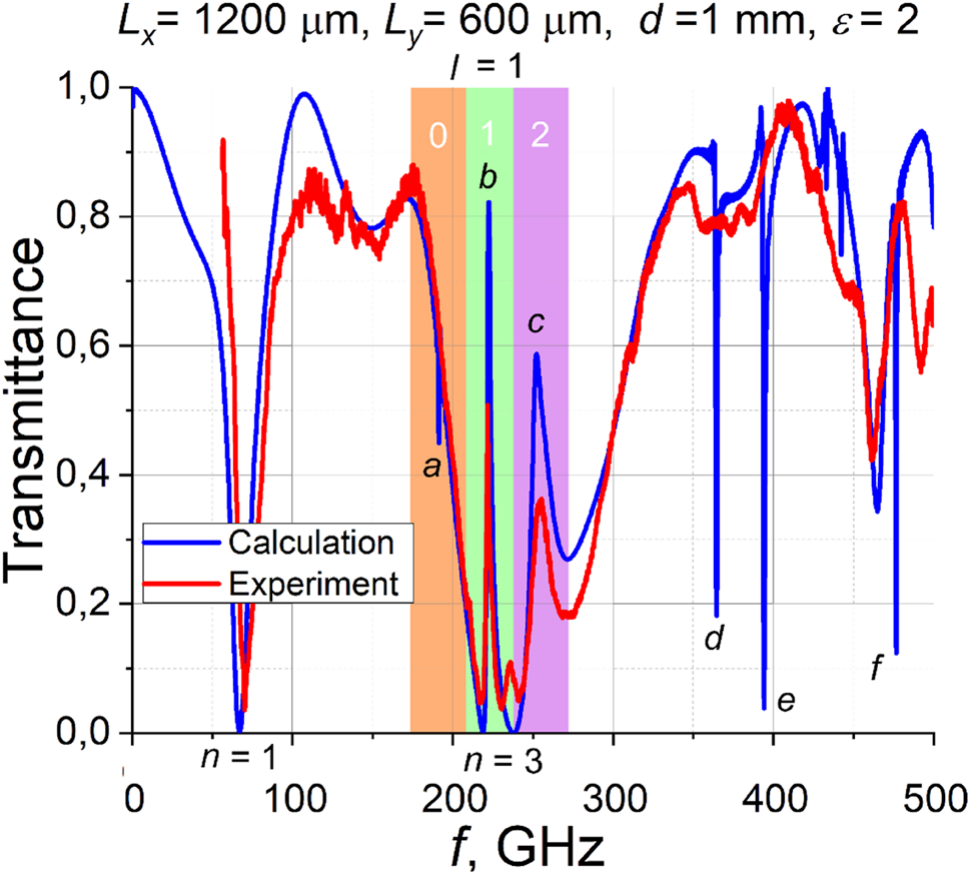
Measured and calculated dependences of transmittance of mirrored SRR on a 1 mm thick PTFE substrate. Letter *n* denotes plasmonic mode number, different colors mark different waveguide modes, numbers in a colored background correspond to waveguide mode, and letter *l* stands for diffraction order. Letters a, b, and c denote Fano resonances.

We measured the transmittance spectrum of SRR arrays deposited on the PTFE substrate to confirm our theoretical consideration. The thickness of the substrate is 1 mm. The SSAIL technology described in Methods is applied. Experimental results together with calculated spectrum are shown in Fig. 6. It is seen that three Fano-type resonances are predicted theoretically. The letters a, b, and c label those resonances. Their calculated Q-factors differ significantly. Very sharp (a) resonance has a Q-factor of more than 200, whereas (b) and (c)—roughly 80 and 30, respectively. As follows from the figure, the sharpest Fano resonance (a) is not distinguished experimentally. Frequency of the measured Fano resonances (b) and (c) perfectly match the simulated data. However, amplitudes of the resonances are lower than predicted, as it usually happens in the THz frequency domain^15^. We found that the amplitudes of the measured Fano peaks are statistically scattered within 10%, due to their high sensitivity to manufacturing technology parameters.

Sharp resonances labeled with the letters d, e, and f have been predicted theoretically with Q-factors ranging from roughly 300 (d) and (e) up to 600 (f). Their characteristic frequencies approximately correspond to m = 0, 1, and 3 modes excited in the substrate due to second-order diffraction on 1200 µm period grating deposited on the surface of 1 mm thick substrate (ref to Fig. 2). Resonances (d), (e) and (f) appear in the transmission spectrum as transmission minima at frequencies where waveguide modes are excited. However, they are not resolved experimentally due to the insufficient accuracy of sample processing.

In view of the results obtained, we have observed for the first time multiple Fano-type resonances appearing in metasurfaces with mirror-oriented resonators formed on sufficiently thick substrates due to the interaction of the waveguide modes with the plasmonic mode. Analysis of the surface currents at resonance (not shown) reveals that the physical reasons for Fano's resonance appearance in the present paper are practically the same as in our previous work, where more details about the dipole moment of currents flowing in the SRR at transmission maximum and minimum can be found^15^.

## Conclusions

As a result of the interaction of plasmonic (n = 3) mode with dielectric waveguide modes, the multiple Fano resonances have been predicted theoretically in a mirrored array of split-ring resonators deposited on a thick PTFE substrate. SSAIL method has been adapted for the formation of high-quality SRRs from copper on a PTFE substrate. Theoretical predictions have been confirmed experimentally on the metasurface manufactured using SSAIL technology. In comparison with^13,14^, where multiple resonances have also been observed, the proposed unit cell of the metasurface is much simpler in the present work. Moreover, the number, frequency, and amplitude of Fano resonances might be adjusted by changing the thickness of the substrate.

## Methods

### Material

Polytetrafluoroethylene (PTFE) was used as a substrate material for SRRs in this work.

### Copper SSRs formation on PTFE substrate using SSAIL method combined with mask photolithography

**Figure 7:**
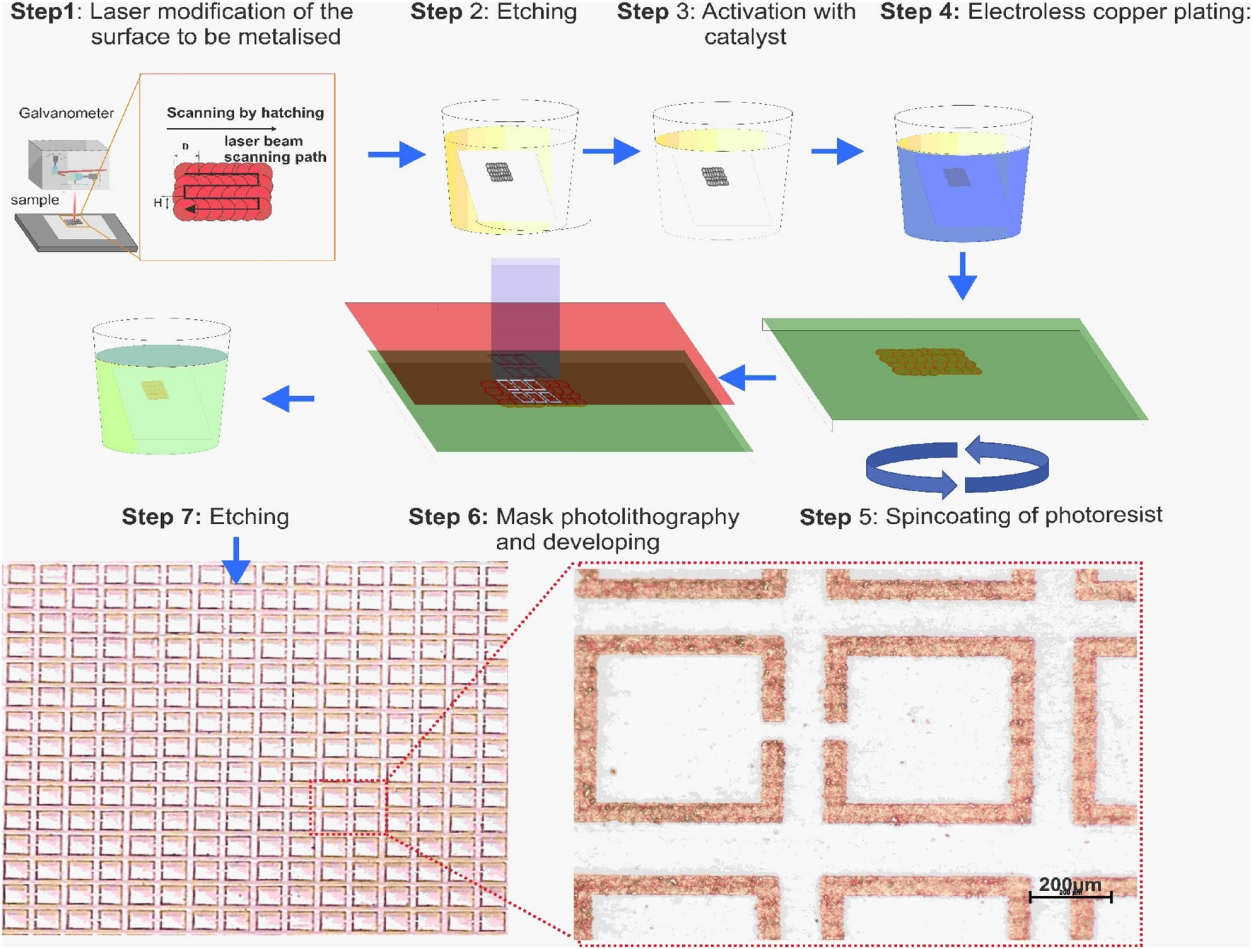
A general diagram of copper deposition on PTFE using SSAIL technology and SRR manufacturing using the standard photomasking method. Step 1—surface treatment with the laser of areas which intend to be metallized, step 2—etching of laser treated areas, step 3—chemical activation of laser treated areas with silver catalyst, step 4—electroless copper plating in an alkaline bath, step 5—spincoating of photoresist, step 6—mask photolithography and developing process, step 7—etching of metasurface structure RCA acid solution and rinsing afterwards.

SSAIL contains three main steps: surface modification by laser beam, chemical activation of the modified areas by dipping into a special solution, and chemical electroless metal deposition on the activated parts. A general diagram of surface activation, its metallization, and SRR manufacturing is shown in Fig. 7.

The laser modification step for selective deposition of copper is performed using the Nd: YVO4 picosecond laser Atlantic (Ekspla). The pulse duration is 10 ps, the repetition rate is 400 kHz–1 MHz, and the maximum average power is up to 60 W. A pulse picker is employed to adjust a lower frequency regime. Translation of the laser beam is performed with a galvanometric scanner (Scanlab AG). The F-theta lens of 160 mm focal length is used to focus the laser beam on the surface of the substrate. The laser beam was scanned over the area to be metallized by hatching—overlapping parallel lines. The focused laser beam spot size was 25 µm in diameter (Gaussian intensity level 1/e^2^).

The specially adapted steps of the SAIL process for the PTFE polymer are the following. After laser writing, the specimen is dipped in Fluoropolymer etch (ARTILABO International BVBA, Belgium) mixture with Toluene for 20–30 s. Next, a highly diluted silver nitride (Sigma-Aldrich) solution is used for the chemical activation of samples. Further, the electroless copper deposition is performed for 60 min at 30 °C. The copper plating bath contains copper sulfate pentahydrate (0.12 M), formaldehyde (0.3 M), sodium hydroxide (1.2 M), sodium carbonate (0.3 M), and sodium–potassium tartrate (0.35) (all Sigma-Aldrich). The pH of the solution is 12.7. When the metallization process is finished, a roughly 10 µm thick layer of copper is formed on the sample's surface. Resonators on the surface of PTFE are fabricated using conventional photolithography and wet etching techniques, including steps: firstly, copper oxides have been removed by emerging samples in 4% by volume acetic acid solution and then dried with a nitrogen gun; further samples were put on hot plate for 10 min 120 °C for water vapor removal, after that samples spin coated with AZ1518 photoresist 30 s 1500 RPM, making 3 µm layer, then heated on hot plate 4 min at 60 °C using low temperature to avoid substrate folding/bending (SUSS MA/BA6 Gen 4 was used for mask alignment), in next step UV source power is set to a constant dose of 100 mJ⁄(cm^2^), exposure configuration 100006249 HR-A-IFP (no Filter 37%). Mask is pressed against the sample using the vacuum contact method. During the mask aligning step, SRR mask is oriented, so SRR symmetry axis (Fig. 1) matches laser hatch markings. Next, samples are washed in developer 1:4 351B:H20 by volume for 1 min; Etching is done with modified RCA solution 30:5:1 H_2_O:HCl:H_2_O_2_ by volume about 2 min, then the photoresist is removed by washing samples in acetone. Final cleaning is performed by washing samples in deionized water and drying with a nitrogen gun. Details of the processes are shown in Fig. 7. The resonators are fabricated on 1 mm thickness PTFE sheets. The lateral size of the investigated samples is 2 × 2 cm^2^. Dimensions of SRR are *A* = 500 μm, *W* = *G* = 50 μm.

### Power transmittance spectra measurement

The power transmitted through the structure is measured using a commercial frequency-domain terahertz spectrometer (Toptica Terascan 780). The electromagnetic wave incidents perpendicularly to the sample plane. The electric field vector is directed in the *y*-direction. Far-field transmittance spectra are obtained by normalizing the measured transmitted power to the reference one, measured in the absence of the investigated sample. The frequency step is kept at 0.2 GHz. In the THz range, dissipative losses are negligible since the metals are almost perfect conductors^33^, and the PTFE substrate is nonabsorbing.

## Data Availability

The datasets analyzed during the current study available from the corresponding author on reasonable request.

## References

[1] Fedotov VA (2007). Sharp trapped-mode resonances in planar metamaterials with a broken structural symmetry. Phys. Rev. Lett..

[2] Singh R (2014). Ultrasensitive terahertz sensing with high-Q Fano resonances in metasurfaces. Appl. Phys. Lett..

[3] Ou H, Lu F, Xu Z, Lin Y-S (2020). Terahertz metamaterial with multiple resonances for biosensing application. Nanomaterials.

[4] Liu S-D (2016). Polarization-independent multiple Fano resonances in plasmonic nonamers for multimode-matching enhanced multiband second-harmonic generation. ACS Nano.

[5] Liu X (2011). Taming the blackbody with infrared metamaterials as selective thermal emitters. Phys. Rev. Lett..

[6] Amarloo H, Hailu DM, Safavi-Naeini S (2014). Multiple Fano resonances structure for terahertz applications. Progress Electromag. Res. Lett..

[7] Born N (2014). Excitation of multiple trapped-eigenmodes in terahertz metamolecule lattices. Appl. Phys. Lett..

[8] Yang L (2018). Characteristics of multiple Fano resonances in waveguide-coupled surface plasmon resonance sensors based on waveguide theory. Sci. Rep..

[9] Chao C-TC, Chau Y-FC, Chiang H-P (2021). Multiple Fano resonance modes in an ultra-compact plasmonic waveguide-cavity system for sensing applications. Results Phys..

[10] Zhao X, Zhu L, Yuan C, Yao J (2016). Reconfigurable hybrid metamaterial waveguide system at terahertz regime. Opt. Express.

[11] Li W (2018). High-Q multiple Fano resonances sensor in single dark mode metamaterial waveguide structure. IEEE Photon. Technol. Lett..

[12] Li W, Lin Q, Zhai X, Wang L (2018). Numerical analysis of high-Q multiple Fano resonances. J. Opt. Soc. Am. B.

[13] Liu S, Xu Z, Yin X, Zhao H (2020). Analog of multiple electromagnetically induced transparency using double-layered metasurfaces. Sci. Rep..

[14] Zhang Z (2021). Single-layer metasurface for ultra-wideband polarization conversion: Bandwidth extension via Fano resonance. Sci. Rep..

[15] Seliuta D, Šlekas G, Valušis G, Kancleris Ž (2019). Fano resonance arising due to direct interaction of plasmonic and lattice modes in a mirrored array of split-ring resonators. Opt. Lett..

[16] Kancleris, Ž., Šlekas, G., Kamarauskas, A., Seliuta, D. Fano resonance in metasurfaces and its application. In *2020 23rd International Microwave and Radar Conference (MIKON)*, Warsaw, Poland, 2020, 328–333, 10.23919/MIKON48703.2020.9253900.

[17] Su CH (2016). Inkjet-printed porphyrinic metal–organic framework thin films for electrocatalysis. J. Mater. Chem. A.

[18] Li M, Li YT, Li DW, Long YT (2012). Recent developments and applications of screen-printed electrodes in environmental assays—A review. Anal. Chim. Acta.

[19] Bariya M (2018). Roll-to-roll gravure printed electrochemical sensors for wearable and medical devices. ACS Nano.

[20] Barreca D (2001). Composition and microstructure of cobalt oxide thin films obtained from a novel cobalt(ii) precursor by chemical vapor deposition. Chem. Mater..

[21] Ratautas K (2019). Laser-assisted selective copper deposition on commercial PA6 by catalytic electroless plating—Process and activation mechanism. Appl. Surf. Sci..

[22] Khairullina EM (2021). High rate fabrication of copper and copper–gold electrodes by laser-induced selective electroless plating for enzyme-free glucose sensing. RSC Adv..

[23] Ratautas K (2020). Evaluation and optimization of the SSAIL method for laser-assisted selective electroless copper deposition on dielectrics. Results Phys..

[24] Ratautas K (2018). Percolation effect of a Cu layer on a MWCNT/PP nanocomposite substrate after laser direct structuring and autocatalytic plating. RSC Adv..

[25] Wallauer J, Bitzer A, Waselikowski S, Walther M (2011). Near-field signature of electromagnetic coupling in metamaterial arrays: A terahertz microscopy study. Opt. Express.

[26] Hubert J (2012). Etching processes of polytetrafluoroethylene surfaces exposed to He and He-O-2 atmospheric post-discharges. Langmuir.

[27] Liu CZ (2004). Comparative study on the effect of RF and DBD plasma treatment on PTFE surface modification. Mater. Chem. Phys..

[28] Taflove A, Hagness SC (1995). Computational Electrodynamics: The Finite-Difference Time–Domain Method.

[29] Šlekas G, Ragulis P, Seliuta D, Kancleris Ž (2017). Using of generalized Goertzel algorithm for FDTD calculation of the transmission and reflection spectra of periodic structures. IEEE Trans. Electromagn. Compat..

[30] Orfanidis SJ (2002). Electromagnetic Waves and Antennas.

[31] Glytsis EN (2020). Introduction to Slab Dielectric Waveguides.

[32] Seliuta D, Šlekas G, Kamarauskas A, Kancleris Ž (2022). Guided lattice modes in terahertz metasurface deposited on ultrathin dielectric substrate. IEEE Trans. Terahertz Sci. Technol..

[33] Singh R (2008). Effect of metal permittivity on resonant properties of terahertz metamaterials. Opt. Lett..

